# Tracking the Trajectory of Resilience: The Impact of Significant Life Events on Resilience in Older Adults

**DOI:** 10.1093/geroni/igaf122.3061

**Published:** 2025-12-31

**Authors:** Jakob Phillips, Jaclyn Bergstrom, Rebecca Daly, Danielle Glorioso, Anthony Molina

**Affiliations:** University of California, San Diego, San Diego, California, United States; University of California, San Diego, San Diego, California, United States; University of California, San Diego, San Diego, California, United States; University of California, San Diego, San Diego, California, United States; University of California, San Diego, San Diego, California, United States

## Abstract

Resilience can change over time and in response to challenging life events, yet the underlying changes in older adulthood and the influence of demographic factors remain unclear. This study examined the 10-year trajectory of resilience in community-dwelling adults and its association with demographic factors and the experience of past-year life events. We analyzed self-report survey data from 1,081 adults in the Successful Aging Evaluation (SAGE) study, a longitudinal study in San Diego County, CA. Resilience was measured using the CD-RISC-10 scale and recent life events with the WHI-LES in all yearly surveys. Linear mixed-effects models assessed associations between recent life events (frequency and perceived impact), demographics, and resilience measured 6 times over 10 years. Results indicated that older age and socioeconomic factors, particularly gender and income, are associated with better resilience trajectories. While resilience showed a small decline with age (b=-0.02, p = 0.008), the decline was greater with a higher number of life events (b=-0.25, p<.001). Notably, the effect of life events on resilience trajectory was not as severe for adults over 65 (b = 0.003, p = 0.017). Household income above $75,000 was associated with greater resilience at baseline (b = 0.89, p = 0.001), underscoring the protective role of economic resources. Men reported significantly higher resilience trajectories than women (b = 0.86, p = 0.012). These findings highlight the dynamic nature of resilience in adulthood and the critical role of socioeconomic factors in fostering stability. Future research should explore protective factors that help sustain resilience across the lifespan and implement interventions to support individuals facing cumulative life challenges.

